# Influence of stage at diagnosis on survival differences for rectal cancer in three European populations

**DOI:** 10.1038/sj.bjc.6690716

**Published:** 1999-10

**Authors:** E Monnet, J Faivre, L Raymond, I Garau

**Affiliations:** Department of Public Health, Faculty of Medicine and Pharmacy, Place Saint Jacques, 25030 Besançon Cedex, France; Côte d'Or Digestive Tract Tumour Registry, Faculty of Medicine, Dijon, France; Geneva Tumour Registry, Geneva, Switzerland; Mallorca Cancer Registry, Universitat de les Illes Balears, Palma de Mallorca, Spain

**Keywords:** rectal neoplasm, staging, survival analysis, registries, international studies

## Abstract

Important differences have recently been highlighted between European countries in the survival of colorectal cancer patients. As data on stage at diagnosis were available for rectal cancers in three European population-based registries (Geneva Switzerland; Côte d'Or, France; Mallorca, Spain), we compared relative survival while assessing the effect of stage in a multiple regression model. We analysed 1005 rectal cancer cases diagnosed between 1982 and 1987 and followed up for at least 5 years. In the Mallorca registry, 16% of the patients were diagnosed in the TNM stage I (versus 21% in the Côte d'Or registry and 29% in the Geneva registry, *P* < 10^−4^) and the 5-year relative survival rate was lower (35%) than in the other two registries (Côte d’Or 47%, Geneva 48%, *P* = 0.01). In the multivariate analysis, stage was the only independent prognostic factor, whereas the excess death risk did not vary significantly among registries (compared to Geneva, Côte d'Or relative risk was 1.0, Mallorca relative risk 1.11, 95% confidence interval 0.76–1.32 and 0.85–1.44 respectively). Survival differences between the registries were mainly due to stage at diagnosis. Thus, diagnostic conditions appear to be the main determinant of the survival inequalities found in those three European populations. © 1999 Cancer Research Campaign


*British Journal of Cancer* (1999) **81**(3), 463–468
© 1999 Cancer Research Campaign
Article no. bjoc.1999.0716

### Influence of stage at diagnosis on survival differences for rectal cancer in three European populations

**E Monnet[1], J Faivre[2], L Raymond[3] and I Garau[4]**

[1]Department of Public Health, Faculty of Medicine and Pharmacy, Place Saint Jacques, 25030 Besançon Cedex, France; [2]Côte d'Or Digestive Tract Tumour Registry, Faculty of Medicine, Dijon, France; [3]Geneva Tumour Registry, Geneva, Switzerland; [4]Mallorca Cancer Registry, Universitat de les Illes Balears, Palma de Mallorca, Spain

**Summary** Important differences have recently been highlighted between European countries in the survival of colorectal cancer patients. As data on stage at diagnosis were available for rectal cancers in three European population-based registries (Geneva Switzerland; Côte d'Or, France; Mallorca, Spain), we compared relative survival while assessing the effect of stage in a multiple regression model. We analysed 1005 rectal cancer cases diagnosed between 1982 and 1987 and followed up for at least 5 years. In the Mallorca registry, 16% of the patients were diagnosed in the TNM stage I (versus 21% in the Côte d'Or registry and 29% in the Geneva registry, $P < 10^{-4}$) and the 5-year relative survival rate was lower (35%) than in the other two registries (Côte d'Or 47%, Geneva 48%, $P = 0.01$). In the multivariate analysis, stage was the only independent prognostic factor, whereas the excess death risk did not vary significantly among registries (compared to Geneva, Côte d'Or relative risk was 1.0, Mallorca relative risk 1.11, 95% confidence interval 0.76–1.32 and 0.85–1.44 respectively). Survival differences between the registries were mainly due to stage at diagnosis. Thus, diagnostic conditions appear to be the main determinant of the survival inequalities found in those three European populations. © 1999 Cancer Research Campaign

**Keywords:** rectal neoplasm; staging; survival analysis; registries; international studies

When dealing with survival data for a potentially curable cancer, population-based cancer registries provide information that can be used to evaluate the performance of health services. For rectal cancers, Eurocare study results have shown important differences between European countries (Coebergh, 1995). Variations in therapy, which have recently been highlighted among countries, may contribute to these inequalities (Gatta et al, 1996). Stage at diagnosis is another important determinant in survival. Nevertheless, it is not routinely collected by all registries and we are faced with specific problems of standardization, thus stage at diagnosis cannot as yet be studied on a European scale.

Because the available data on rectal cancer stage were very accurate and covered a long period of time in the three European population-based registries (Geneva, Switzerland; Côte d'Or, France; Mallorca, Spain), it was possible to create a common classification consistent with TNM staging (Sobin et al, 1997). The aim of this study was to compare relative survival after rectal cancer in those three European areas while assessing the effect of stage at diagnosis.

### PATIENTS AND METHODS

The population registries included in the study were the general Cancer Registry of Geneva (GCR) Switzerland, and the digestive tract Cancer Registries of Côte d'Or (COCR) France and Mallorca (MCR) Spain. The populations covered by the registry were similar in size (for 1985, 371 400 inhabitants in the canton of Geneva, 473 700 in Côte d'Or and 550 800 in Mallorca). During the study period, which extended from 1982 (when registration began in Mallorca) to 1987, 1456 incident cases of rectal cancers (within 15 cm from the anal verge, ICD-O 1540–1548) were registered in the three populations. The world age-standardized incidence rates in males ranged from 12.4 in Mallorca to 15.6 per 100 000 in Côte d'Or, and in females from 7.0 in Mallorca to 9.1 per 100 000 in the canton of Geneva.

The proportion of cases notified by death certificate alone (DCO) ranged from 0% in the COCR to 6% in the MCR, and the proportion of histologically verified cases from 96% in the MCR (after excluding DCO cases) to 98% in the COCR.

The study was restricted to 1148 patients under 80 years of age, because, past that age, there were disparities between the registries in the ability both to diagnose and to register cancer cases (Monnet et al, 1998). Patients with anal cancer (ICD-9 1543: 58 patients), rectal lymphoma or sarcoma (14 patients), DCO cases (22 patients) and cases discovered at autopsy (eight patients) were not included.

### Studied variables

The three registries routinely collected detailed clinical data, such as tumour extension and type of treatment, from hospital records, operative reports and pathology reports. As the staging procedures used at the time in each registry were different, we created a classification algorithm taking into account the primary treatment received by the patients. Patients whose data on primary

*Received 26 June 1998*
*Revised 15 April 1999*
*Accepted 21 April 1999*

*Correspondence to:* E Monnet treatment were missing were excluded from the stage classification (GCR 16 patients COCR five patients). Primary treatment was recorded by three registries as follows:

1. surgery for cure (macroscopic resection of all tumoural tissue with no microscopic evidence of proximal and distal margins involvement)
2. palliative surgery (palliative resection, bypass or colostomy)
3. no surgery.

If a patient had surgery for cure, he was classified according to pathology report data in one of the four following classes of the TNM classification: stage I (T1–T2, N0, M0), stage II (T3–T4, N0, M0), stage III (N1–2, M0) or resected stage IV (M1, if metastases were completely removed surgically). Patients with surgery for cure, whose histological results were not available, were classified as stage unknown. In the absence of surgery for cure, patients were classified as not resected stage IV if a visceral metastasis was diagnosed (M1), loco-regional in case of palliative surgery without visceral metastasis (T4, NX, M0), or undetermined in both cases of no surgery and no detected metastasis (TX, NX, M0).

### Statistical analysis

The characteristics of the patients and the distribution of primary treatment and tumour stage at diagnosis were compared between registries with the $\chi^2$ test. Survival was studied for the first 5 years after the date of diagnostic confirmation. Follow-up data were actively collected in the three registries by reviewing death certificates and through contact with hospitals and patients' physicians. The closing date to determine living or dead status was 31 December 1992. On this date, vital status was available for 99% of the patients in the COCR and 95% of the patients in the MCR. For the GCR, the statistical analysis was restricted to Swiss national patients (vital status available for 98% of the patients) because of the high rate of loss to follow-up for foreigners in the Geneva canton (Raymond et al, 1995). Foreigners represented 41 patients among the GCR cases. We computed relative survival rates (Hakulinen, 1982) by using the population life tables established for the Eurocare study corresponding exactly to the geographical area of the registries (Micheli et al, 1995). Relative survival provides an estimate of patients' survival which is corrected for the effect of the causes of death independent of rectal cancer itself. It is defined by the ratio of the observed survival of cancer patients to the survival of an age, sex, geographic area and period matched cohort estimated from population life tables. We compared 5-year relative survival rates according to age at diagnosis (categorized into three groups: under 50 years, 50–64 years, 65–79 years), sex, registry and tumour stage at diagnosis by using the maximum likelihood ratio test (Hakulinen et al, 1987*a*). Then we used a multiple regression model (Hakulinen et al, 1987*b*) to evaluate simultaneously the effects of different prognostic factors on relative survival. In this model, patient mortality hazard is set as an addition to the expected mortality for demographically similar individuals in general population and to the disease-related mortality hazard which represents an excess death risk. For this latter, a proportional hazards model for prognostic covariates is assumed. In this analysis, the 'follow-up' period was divided into five intervals of 1 year each, and the model was fitted with a forward selection of variables. The significance of covariates was tested on the change in deviance. Interaction terms between significant covariates were systematically tested, as were interactions between years of follow-up and prognostic factors in order to study the proportionality of hazards within the time period.

Analyses were performed on IBM compatible microcomputer with the BMDP software (University of California Press, Los Angeles, CA, USA) and the Hakulinen et al programme (Hakulinen et al, 1985) using a Glim macro (Baker et al, 1978).

**Table 1** Rectal cancer cases in each registry by age, sex, primary treatment and stage at diagnosis

| | Registry *n* (%) | | | | | | |
|---|---|---|---|---|---|---|---|
| | Geneva | | Côte d'Or | | Mallorca | | *P*[a] |
| Age (years) | | | | | | | |
| <50 | 15 | (7) | 24 | (6) | 27 | (6) | 0.97 |
| 50–64 | 66 | (31) | 128 | (33) | 137 | (34) | |
| 65–79 | 131 | (62) | 234 | (61) | 243 | (60) | |
| Sex | | | | | | | |
| Male | 106 | (50) | 260 | (67) | 248 | (61) | $10^{-4}$ |
| Female | 106 | (50) | 126 | (33) | 159 | (39) | |
| Primary treatment | | | | | | | |
| Surgery for cure | 152 | (72) | 287 | (74) | 297 | (73) | 0.29 |
| Palliative surgery | 36 | (17) | 60 | (16) | 80 | (20) | |
| No surgery | 24 | (11) | 39 | (10) | 30 | (7) | |
| Stage[b] | | | | | | | |
| I ($T_1$–$T_2$ $N_0$ $M_0$) | 61 | (29) | 81 | (21) | 66 | (16) | $<10^{-4}$ |
| II ($T_3$–$T_4$ $N_0$ $M_0$) | 47 | (22) | 110 | (29) | 102 | (25) | |
| III ($N_1$–$N_2$ $M_0$) | 38 | (18) | 93 | (24) | 101 | (25) | |
| IV ($M_1$) resected | 5 | (2) | 2 | (1) | 12 | (3) | |
| Loco-regional ($T_4$–$N_x$ $M_0$) | 9 | (4) | 11 | (3) | 29 | (7) | |
| Undertermined ($T_x$ $N_x$ $M_0$) | 9 | (4) | 23 | (6) | 13 | (3) | |
| IV ($M_1$) not resected | 42 | (20) | 60 | (16) | 68 | (17) | |
| Unknown[c] | 1 | (0) | 1 | (0) | 16 | (4) | |

[a]Pearson $\chi^2$; [b]five patients from Côte d'Or exclusively treated by contact radiotherapy were excluded from stage classification; [c]patients with surgery for cure and unknown histological results.

 © 1999 Cancer Research Campaign

**Table 2** Crude and relative survival by age, sex, stage and registry

| | Survival rates (s.d.) | | | | | | |
|---|---|---|---|---|---|---|---|
| | One year | | Two years | | Five years | | |
| | Crude | Relative | Crude | Relative | Crude | Relative | $P^a$ |
| Age (years) | | | | | | | |
| <50 | 0.68 (0.06) | 0.68 (0.06) | 0.53 (0.06) | 0.53 (0.06) | 0.32 (0.06) | 0.33 (0.06) | 0.89 |
| 50–64 | 0.77 (0.02) | 0.77 (0.02) | 0.64 (0.03) | 0.66 (0.03) | 0.43 (0.03) | 0.46 (0.03) | |
| 65–79 | 0.70 (0.02) | 0.73 (0.02) | 0.55 (0.02) | 0.60 (0.02) | 0.33 (0.02) | 0.41 (0.02) | |
| Sex | | | | | | | |
| Male | 0.72 (0.02) | 0.74 (0.02) | 0.57 (0.02) | 0.62 (0.02) | 0.34 (0.02) | 0.42 (0.02) | 0.97 |
| Female | 0.73 (0.02) | 0.74 (0.02) | 0.59 (0.02) | 0.62 (0.02) | 0.38 (0.02) | 0.43 (0.03) | |
| Stage[b] | | | | | | | |
| I ($T_1$–$T_2$ $N_0$ $M_0$) | 0.91 (0.02) | 0.94 (0.02) | 0.86 (0.02) | 0.91 (0.03) | 0.67 (0.03) | 0.79 (0.04) | $<10^{-4}$ |
| II ($T_3$–$T_4$ $N_0$ $M_0$) | 0.91 (0.02) | 0.93 (0.02) | 0.82 (0.02) | 0.88 (0.03) | 0.53 (0.03) | 0.63 (0.04) | |
| III ($N_1$–$N_2$ $M_0$) | 0.81 (0.03) | 0.83 (0.03) | 0.57 (0.03) | 0.61 (0.03) | 0.25 (0.03) | 0.28 (0.03) | |
| Loco-regional ($T_4$ $N_x$ $M_0$) | 0.39 (0.07) | 0.40 (0.07) | 0.24 (0.07) | 0.26 (0.07) | 0.04 (0.03) | 0.05 (0.03) | |
| Undetermined ($T_x$ $N_x$ $M_0$) | 0.47 (0.08) | 0.48 (0.07) | 0.28 (0.07) | 0.30 (0.07) | 0.16 (0.06) | 0.20 (0.07) | |
| IV ($M_1$) | 0.27 (0.03) | 0.28 (0.04) | 0.07 (0.01) | 0.07 (0.02) | 0.01 (0.01) | 0.01 (0.02) | |
| Registry | | | | | | | |
| Geneva | 0.74 (0.03) | 0.76 (0.03) | 0.61 (0.03) | 0.65 (0.03) | 0.41 (0.03) | 0.48 (0.04) | 0.01 |
| Côte d'Or | 0.73 (0.02) | 0.76 (0.02) | 0.62 (0.02) | 0.66 (0.03) | 0.40 (0.03) | 0.47 (0.03) | |
| Mallorca | 0.70 (0.02) | 0.72 (0.02) | 0.53 (0.02) | 0.56 (0.03) | 0.29 (0.02) | 0.35 (0.03) | |

[a]Maximum likelihood ratio test comparing relative survival rates. [b]Survival rates were not calculated for stage IV resected and stage unknown because of insufficient number.

For all statistical tests, *P*-values less than or equal to 0.05 were regarded as significant.

### RESULTS

Neither age distribution nor primary treatment were significantly different between the registries (Table 1). The proportion of patients resected for cure was similar in the three registries (GCR 72%, COCR 74%, MCR 73%). On the other hand, there was a significant difference between the registries in sex ratio and distribution of tumour stage at diagnosis. In the GCR, 61 patients (29%) were diagnosed in the TNM stage I, versus 81 (21%) in the COCR and 66 (16%) in the MCR ($P < 10^{-4}$).

Relative survival rates for each class of the studied variables are presented in Table 2. In univariate analysis, age and sex did not have a significant effect on survival, unlike stage at diagnosis which had an important prognostic effect. Relative survival rates varied according to registries: survival was lower in the MCR (35% at 5 years) than in the other two registries (respectively 48% in the GCR and 47% in the COCR $P = 0.01$). Five-year relative survival rates, by stage and site in the three registries, are presented in Table 3. Differences in survival rates between registries were slight for stages II, III and IV. On the other hand, there were greater differences for stage I.

The successive steps to fit the data, when using multiple regression model for relative survival, are presented in Table 4. After including the 'follow-up' effect, stage was the only variable which significantly improved the fit of the model. Age, sex and registry had no significant effect. The relative risk estimates for covariates with their 95% confidence intervals (CIs) are shown in Table 5. Stage at diagnosis had a strong effect on the excess death risk which markedly increased with advancing cancer extension. While adjusting on stage, age and sex, the excess death risk in the MCR remained higher than in the other two registries but the difference was no longer significant.

There was a significant interaction between years of follow-up and stage (Table 4), showing that hazards were not proportional across stage classes for the 5 years of follow-up. For undetermined and metastases stage patients, the relative excess death risks were 3.7 as high for the first 2 years of follow-up as for the subsequent years (95% CI 1.9–7.3). For stage III patients, it was twice as high for the second year as for the other years of follow-up (95% CI 1.3–3.2).

### DISCUSSION

Our results confirm the existence of a survival difference among European countries for rectal cancer (Coebergh, 1995), while

© 1999 Cancer Research Campaign

**Table 3** Five-year crude and relative survival rates by stage in the three registries

| | Five-year survival rates (s.d.) | | | | | |
|---|---|---|---|---|---|---|
| | Geneva | | Côte d'Or | | Mallorca | |
| | Crude | Relative | Crude | Relative | Crude | Relative |
| Stage[a] | | | | | | |
| I ($T_1$–$T_2$ $N_0$ $M_0$) | 0.75 (0.06) | 0.87 (0.06) | 0.68 (0.05) | 0.80 (0.06) | 0.60 (0.06) | 0.68 (0.08) |
| II ($T_3$–$T_4$ $N_0$ $M_0$) | 0.57 (0.07) | 0.67 (0.08) | 0.57 (0.05) | 0.68 (0.06) | 0.46 (0.05) | 0.54 (0.06) |
| III ($N_1$–$N_2$ $M_0$) | 0.26 (0.07) | 0.29 (0.08) | 0.25 (0.05) | 0.29 (0.05) | 0.23 (0.04) | 0.27 (0.05) |
| IV ($M_1$) | 0.00 (0.00) | 0.00 (0.00) | 0.02 (0.02) | 0.02 (0.02) | 0.02 (0.02) | 0.02 (0.02) |

[a]Survival rates were not calculated for stages IV resected, loco-regional ($T_4$ $N_x$ $M_0$), undetermined ($T_x$ $N_x$ $M_0$) and for stage unknown because of insufficient number.

**Table 4** Regression analysis of relative survival rates in rectal cancer: step-wise procedure for testing the covariate significance

| Model | Regressor | Deviance | df | *P*-value |
|---|---|---|---|---|
| 1 | follow-up years | 870 | 370 | |
| 2 | model 1 + stage | 403 | 365 | <0.001 |
| 3 | model 2 + age | 399 | 363 | >0.10 |
| 4 | model 3 + sex | 397 | 362 | >0.30 |
| 5 | model 4 + registry | 396 | 360 | >0.50 |
| 6 | model 2 + stage follow-up[a] | 379 | 363 | <0.001 |

df, degrees of freedom. [a]Model adjusted by including two interaction terms: (1) first 2 years of follow-up and stages undetermined and IV; (2) second year of follow-up and stage III.

**Table 5** Relative excess death risks in rectal cancer (model 5).

| Variable | Relative risk | 95% confidence interval |
|---|---|---|
| Age (years) | | |
| <50 | 1 | |
| 50–64 | 0.76 | 0.52–1.10 |
| 65–79 | 0.94 | 0.66–1.35 |
| Sex | | |
| Male | 1 | |
| Female | 1.12 | 0.91–1.37 |
| Stage | | |
| I ($T_1$–$T_2$ $N_0$ $M_0$) | 1 | |
| II ($T_3$–$T_4$ $N_0$ $M_0$) | 1.88 | 1.16–3.03 |
| III ($N_1$–$N_2$ $M_0$) | 5.23 | 3.36–8.13 |
| Loco-regional ($T_4$ $N_x$ $M_0$) | 14.10 | 8.31–23.83 |
| Undetermined ($T_x$ $N_x$ $M_0$) | 8.80 | 5.02–15.43 |
| IV ($M_1$) | 25.40 | 16.10–40.10 |
| Registry | | |
| Geneva | 1 | |
| Côte d'Or | 1.00 | 0.76–1.32 |
| Mallorca | 1.11 | 0.85–1.44 |

clarifying its possible origins. Differences in both stage at diagnosis and treatment access and quality have been put forward in Eurocare studies (Sant et al, 1995; Gatta et al, 1996). In our study, it is the important difference of stage at diagnosis which mainly explains survival inequalities. Patients in the MCR, less often diagnosed in stage I, have a worse survival than patients in the other two registries. After controlling for stage, survival difference is no longer significant.

Our study provides evidence for the main role of diagnostic conditions in survival inequalities. Up until now, there was no organized screening programme in the three areas. In the GCR, 29% of the patients were diagnosed in stage I. This could be due to both a better education of patients and a better access to early endoscopy in this high standard of living urban area. On the other hand, no variation in therapy efficiency seemed to be involved. There was no significant difference between the registries concerning surgical resection frequency. The reduction of survival difference after adjusting on stage argues in favour of the absence of important variations in treatment outcomes between the three areas. In our study, stage-specific survival rates were lower than the ones observed among patients treated by optimized surgical procedures in specialized centres (MacFarlane et al, 1993; Arbman et al, 1996). Series collected by cancer registries have the major advantage of collecting all the cases diagnosed in a well-defined population, avoiding the selection bias of hospital-based series. Variations of treatment outcomes depending on surgical skills and hospital performances are very plausible within each of the three areas as reported in other populations (McArdle et al, 1991; Holm et al, 1997; Simons et al, 1997).

In international survival comparisons, bias may be caused by several methodological problems (Berrino et al, 1995) and comparability of data in each population needs to be investigated. This was performed in a preliminary work (Monnet et al, 1998), which showed the high completeness and validity of rectal cancer data in

 *© 1999 Cancer Research Campaign*

the three registries for patients up to 79 years. Therefore no issue of patient selection, definition of the disease, follow-up system or method of calculating survival duration may explain our results.

In this population study, as in other published series, stage at diagnosis was the major prognostic factor. Stage-specific survival rate and relative risk estimates were close to those reported in other populations (Kune et al, 1990; Arbman et al, 1995; Roncucci et al, 1996). Relative risks, as shown in Table 4, have to be considered as average estimates since the hazards were non-proportional across stage classes for the 5 years of follow-up. In advanced stages, the excess death risks were maximum for the first 2 years and decreased thereafter, while the excess death risk of stage III patients was more marked for the second year. The classification used in our study was consistent with TNM staging, based on histological examinations and took into account the primary treatment received by patients. In colorectal cancers, staging and surgical treatment are interrelated procedures (Kronborg, 1993). Our classification was stratified with surgical treatment modalities in order to reduce the differences in stage measure conditions between the registries. Indeed, as they depend on diagnostic technology and medical practice, stage measure conditions vary with time and place (Feinstein et al, 1985). The slightly higher frequency of metastases in the GCR could be due to a more thorough exploration of patients in the Geneva canton. As pointed out by several authors (Blenkinsopp et al, 1981; Bull et al, 1997) the quality of routine pathology data noticeably varies between laboratories. It depends in particular on the thoroughness of the examination and on the completeness of lymph node resection. These criteria could not be analysed in our study and we assume the data were of similar quality, on average, in the three areas. Our results cannot be explained by an information bias since there is a survival difference between the registries in univariate analysis.

The poorer prognosis in young patients, when compared with older ones, is still debated (Smith et al, 1989; Isbister et al, 1990; Enblad et al, 1990). In our study, after controlling for stage, patients under 50 tended to have a lower survival than older patients, but the difference did not become significant. Moreover, poor prognosis stages were more frequent among patients under 50 than among older ones: before 50 years, 33% of patients were diagnosed with lymph node metastases and 23% with visceral metastases versus, respectively, 22% and 17% of patients aged 50 years and over ($P = 0.02$).

Results from population-based studies reveal that rectal cancer prognosis is highly correlated to the health service ability to provide all patients with both an early diagnosis and treatment in specialized centres. By studying the effect of tumour stage at diagnosis, our work highlights the importance of access to diagnostic examinations. A delay in diagnosis, particularly among younger patients, leads to more advanced and less curable tumours. The determinants of access to diagnosis are numerous and complex. They include endoscopy availability and financing, practitioner education as well as population information. Further studies are required to investigate the role of health care supply and organization and to compare practice standards.

Rectal cancer outcomes in leading populations, such as the canton of Geneva, could be considered as an attainable objective by public health authorities in less advanced countries. Current differences between European populations suggest that health benefits within health policies' reach could be greater in many countries than that of any of the adjuvant therapies currently under study.

### ACKNOWLEDGEMENTS

The authors thank R Capocaccia, Instituto Superiore Di Sanita, Laboratorio di Epidemiologia, Rome, Italy for providing the Eurocare life tables.

### REFERENCES

Arbman G, Nilsson E, Störgren-Fordell V and Sjödahl R (1995) Outcome of surgery for colorectal cancer in a defined population in Sweden from 1984 to 1986. *Dis Colon Rectum* **38**: 645–650

Arbman G, Nilsson E, Hallböök O and Sjödahl R (1996) Local recurrence following total mesorectal excision for rectal cancer. *Br J Surg* **83**: 375–379

Baker RJ and Nelder JA (1978) The Glim System, Release 3. Generalized linear interactive modelling. Numerical Algorithms group: Oxford

Berrino F, Estève J and Coleman MP (1995) Basic issues in estimating and comparing the survival of cancer patients. In: *Survival of Cancer Patients in Europe: The Eurocare Study*, Berrino F, Sant M, Verdecchia A, Capocaccia R, Hakulinen T and Estève J (eds), pp. 1–14. IARC scientific publication no. 132, IARC: Lyon

Blenkinsopp WK, Stewart-Brown S, Blesovsky L, Kearney G and Fielding LP (1981) Histopathology reporting in large bowel cancer. *J Clin Pathol* **34**: 509–513

Bull AD, Biffin AH, Mella J, Radcliffe AG, Stamatakis JD, Steele RJ and Williams GT (1997) Colorectal cancer pathology reporting: a regional audit. *J Clin Pathol* **50**: 138–142

Coebergh JWW (1995) Summary and discussion of results. In: *Survival of Cancer Patients in Europe: The Eurocare Study*, Berrino F, Sant M, Verdecchia A, Capocaccia R, Hakulinen T and Estève J (eds), pp. 447–463. IARC scientific publication no. 132, IARC: Lyon

Enblad G, Enblad P, Adami HO, Glimelius B, Krusemo V and Pählman L (1990) Relationship between age and survival of the colon and rectum with special reference to patients less 40 than years of age. *Br J Surg* **77**: 611–616

Feinstein AR, Sosin DM and Wells CK (1985) The Will Rogers phenomenon. Stage migration and new diagnostic techniques as a source of misleading statistics for survival in cancer. *N Engl J Med* **312**: 1604–1608

Gatta G, Sant M, Coebergh JW, Hakulinen T and the Eurocare Working Group (1996) Substantial variation in therapy for colorectal cancer across Europe: Eurocare analysis of cancer registry data for 1987. *Eur J Cancer* **32A**: 831–835

Hakulinen T (1982) Cancer survival corrected for heterogeneity in patient withdrawal. *Biometrics* **38**: 933–942

Hakulinen T and Abeywickrama KH (1985) A computer program package for relative survival analysis. *Comp Progr Biomed* **19**: 197–207

Hakulinen T and Tenkanen L (1987) Regression analysis of the relative survival rates. *Appl Stat* **36**: 309–317

Hakulinen T, Tenkanen L, Abeywickrama KH and Päivärinta (1987) Testing equality of relative survival patterns based on aggregated data. *Biometrics* **43**: 315–325

Holm T, Johansson H, Cedermark B and Ekelund G (1997) Influence of hospital- and surgeon-related factors on outcome after treatment of rectal cancer with or without preoperative radiotherapy. *Br J Surg* **84**: 657–663

Isbister WH and Fraser J (1990) Large bowel cancer in the young. A national survival study. *Dis Colon Rectum* **33**: 363–366

Kronborg O (1993) Staging and surgery for colorectal cancer. *Eur J Cancer* **29A**: 575–583

Kune GA, Kune S, Field B, White RG, Brough W, Schellenberger R and Watson LF (1990) Survival in patients with large bowel cancer. A population-based investigation from the Melbourne colorectal cancer study. *Dis Colon Rectum* **33**: 938–946

McArdle CS and Hole D (1991) Impact of variability among surgeons on postoperative morbidity and mortality and ultimate survival. *Brit Med J* **302**: 1501–1505

MacFarlane JK, Ryall RDH and Heald RJ (1993) Mesorectal excision for rectal cancer. *Lancet* **341**: 457–460

Micheli A and Capocaccia R (1995) General mortality and its effect on survival estimates. In: *Survival of Cancer Patients in Europe: The Eurocare Study*, Berrino F, Sant M, Verdecchia A, Capocaccia R, Hakulinen T and Estève J (eds), pp. 38–46. IARC scientific publication no. 132, IARC: Lyon

Monnet E, Faivre J, Raymond L and Garau I (1998) Comparability of colorectal cancer survival data in three European population-based registries. *Eur J Cancer Prev* **7**: 127–134

Raymond L and Torhost J (1995) Health care system, cancer registration and follow-up of cancer patients in Switzerland. In: *Survival of Cancer Patients in*

*© 1999 Cancer Research Campaign*

*Europe: The Eurocare Study*, Berrino F, Sant M, Verdecchia A, Capocaccia R, Hakulinen T and Estève J (eds), pp. 69–70. IARC scientific publication no. 132, IARC: Lyon

Roncucci L, Fante R, Losi L, Di Gregorio C, Micheli A, Benatti P, Madenis N, Ganazzi D, Cassinadri MT, Lauriola P and Ponz de Leon M (1996) Survival for colon and rectal cancer in a population-based cancer registry. *Eur J Cancer* **32A**: 295–302

Sant M, Capocaccia R, Verdecchia A, Gatta G, Micheli M, Mariotto A, Hakulinen T, Berrino F and the Eurocare Working Group (1995) Comparisons of colon cancer survival among European countries: the Eurocare study. *Int J Cancer* **63**: 43–48

Simons AJ, Ker R, Groshen S, Anthone GJ, Ortega AE, Vukasin P, Ross RK and Beart RW (1997) Variations in treatment of rectal cancer: the influence of hospital type and caseload. *Dis Colon Rectum* **40**: 641–646

Smith C and Butler JA (1989) Colorectal cancer in patients younger than 40 years of age. *Dis Colon Rectum* **32**: 843–846

Sobin LH and Wittekind Ch (1997) *International Union Against Cancer TNM Classification of Malignant Tumors*, 5th edn. John Wiley & Sons: New York

*© 1999 Cancer Research Campaign*
